# T77. DIAGNOSTIC AND NEUROCOGNITIVE CORRELATES OF SCHIZOTYPY WITHIN AND ACROSS THE PRONIA STUDY GROUPS

**DOI:** 10.1093/schbul/sby016.353

**Published:** 2018-04-01

**Authors:** Carolina Bonivento, Maria Fernanda Urquijo, Stefan Borgwardt, Eva Meisenzahl, Marlene Rosen, Raimo Salokangas, Rachel Upthegrove, Stephen Wood, Nikolaos Koutsouleris, Paolo Brambilla

**Affiliations:** 1 University of Udine; 2 Ludwig-Maximilian-University; 3 University of Basel; 4 Heinrich-Heine University Düsseldorf; 5 University of Cologne; 6 University of Turku; 7 University of Birmingham; 8 The University of Melbourne; 9 University of Milan

## Abstract

**Background:**

Schizotypy traits range from odd behaviors to symptoms that resemble full schizophrenia, although less severe. Previous studies associated different degrees of positive and negative schizotypal traits to variations in the persons’ cognitive profiles while others related them to the risk to develop psychosis.

We hypothesize that similar pattern of positive and negative schyzoypy traits characterize individuals at risk of psychosis and patients meeting the criteria for recent onset psychosis, although with different degrees of severity. Also, both should differ from depressed patients. Moreover, specific combinations of schizotypy traits and neurocognitive alterations should be associated to the different psychopathological profiles. The final goal of the study is to identify candidate predictors of risk of psychosis that will be used as features in next machine learning analyses.

**Methods:**

The present is a multi-centric study that was conducted as part of the project titled ‘Personalised Prognostic Tools for Early Psychosis Management’ (PRONIA).

115 participants at high-risk for psychosis (CHR), 114 recent onset psychosis (ROP), 123 recent onset depression (ROD) and 252 healthy controls (HC) took part in the study.

All were aged between 15 and 40 years.

The participants filled the Wisconsin Schizotypy questionnaire, measuring positive (Magical Ideation Scale - MIS; Perceptual Aberration Scale - PAS) and negative schizotypy traits (Social Anhedonia Scale - SAnS; Physical Anhedonia Scale - PAnS).

Moreover, they were administered the PRONIA Cognitive Battery (PCB), comprising measures of visuo-spatial dexterity and memory (Rey Figure, copy and delayed drawing), short-term memory (Digit Span - DS), Verbal Learning, Verbal Fluency, Attention (Continuous Performance Test - CPT, Digit Symbol Substitution Test - DSST), Emotions’ Recognition, General Intelligence (WAIS Vocabulary, Matrix Reasoning).

**Results:**

We run i) a Multivariate Analysis of Covariance with ‘WSS subscales’ as dependent variable; ‘Group’ as between subject factor; ‘Age’ and ‘Gender’ as covariates; ii) a Multinomial logistic regression with ‘Group’ as dependent variable; HC ‘Group’ as reference parameter; ‘WSS subscales’ and scores at the PCB’s tests as predictors; ‘Age’ and ‘Gender’ as covariates.

ROP and CHR reported both positive and negative schizotypy traits, although only the negative symptoms involving social aspects were clearly evident in CHR. Also, ROP and CHR differed for the positive symptoms, as they were present but at a lower level in CHR than in ROP. ROD instead scored high at the negative symptoms. Interestingly, ROP, CHR and ROD did not differ between each other for the negative symptoms, probably reflecting the effect of the psychopathology on the patients’ general motivation to life.

The regressions analysis highlighted different patterns of associations of WSS and neurocognitive scores with the clinical status. In particular, the scores at the MIS, PAS and SanS combined with the Rey Figure (delayed drawing), predicted that the participants were CHR; the MIS, PAS and SAnS with measures of attention (CPT, DSST) predicted that the participant were ROP; the PAS; SAnS and short-term memory (DS) predicted to being ROD.

**Discussion:**

Coherently with the hypotheses, different schizotypy traits or grade of severity characterized patients with distinct psychopathology profiles. Also, the association of WSS subscales with the cognitive measures differentiated between groups, with visuo-spatial long-term memory being associated to CHR, measures of attention relating to ROP and verbal short term memory relating to ROD.

This makes these measures good candidates for the upcoming machine learning analyses.

